# Community Care Stakeholders’ Reflection on Using Telepresence Robots as a Tool to Reduce School Absence for Children and Young People with Chronic Illness in the Scandinavian Municipalities

**DOI:** 10.3390/ijerph23060743

**Published:** 2026-06-01

**Authors:** Sofie Skoubo, Hanne Bækgaard Larsen, Mette Weibel Willard, Charlotte Handberg

**Affiliations:** 1 National Rehabilitation Center for Neuromuscular Diseases, 8000 Aarhus, Denmark; chha@rcfm.dk; 2 Department of Public Health, Faculty of Health, Aarhus University, 8000 Aarhus, Denmark; 3 Department of Pediatrics and Adolescent Medicine, The Juliane Marie Center, Copenhagen University Hospital, 2100 Copenhagen, Denmark; hanne.baekgaard.larsen@regionh.dk (H.B.L.); mww@boernecancerfonden.dk (M.W.W.); 4 Department of Clinical Medicine, Faculty of Health and Medical Sciences, University of Copenhagen, 2200 Copenhagen, Denmark

**Keywords:** chronic illness, telepresence robots, management, stakeholder reflections, school absence, interpretive description, organizational culture, students

## Abstract

**Highlights:**

**Public health relevance—How does this work relate to a public health issue?**

- School absence affects social participation, educational development, and long-term health outcomes for students with chronic illness.
- Telepresence robots can be an educational tool to enhance participation in the education environment.

**Public health significance—Why is this work of significance to public health?**

- A telepresence robot can promote participation and educational continuity during school absence for students with chronic illness.
- The study presents an educational support tool to address inclusion and equal opportunity in the education system.

**Public health implications—What are the key implications or messages for practitioners, policy makers and/or researchers in public health?**

- Practitioners, policy makers, and healthcare professionals should include telepresence robots as an educational tool to support children with chronic illness in their educational participation to prevent long-term social and educational disadvantages.
- Findings underscore the need for clear guidelines, policy decisions, and coordination to include telepresence robots as an educational tool in the education system in Scandinavia.

**Abstract:**

Chronic illness negatively affects education due to school absence. Telepresence robots can be a valuable educational tool for reducing school absence among students with chronic illnesses. This study aimed to investigate the reflections of Scandinavian community care stakeholders on telepresence robots as a tool to reduce school absence for students with chronic illnesses in the education system. We conducted fifteen semi-structured interviews and four focus group interviews with 25 community care stakeholders. Our study used the interpretive description methodology and Edgar Schein’s organizational culture as the theoretical lens. The analysis identified three categorical themes and six subthemes: The action of integrating telepresence robots into the education system, the telepresence robots as a pathway to educational opportunities, and the adoption and management of telepresence robots. Our findings showed that stakeholders had to motivate and communicate the purpose of telepresence robots to reduce skepticism among multiple people in the school environment. Our study provides insights into the barriers and challenges to integrating and adopting telepresence robots in the Scandinavian education systems, as well as how stakeholders should communicate with and support the education system during their implementation.

## 1. Introduction

**Figure 1:**
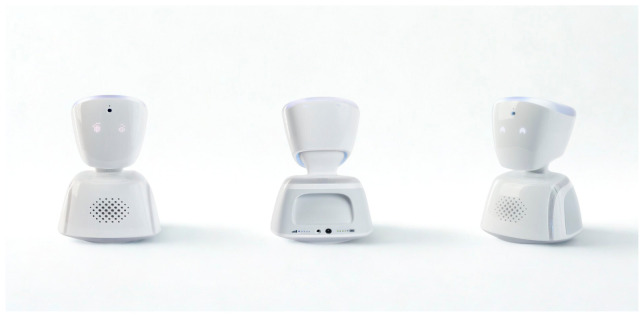
The AV1 telepresence robot (No Isolation, Oslo, Norway).

Education can ensure better health and prevent unemployment and social exclusion [1,2]. Chronic illness serves as an umbrella term for various physical and mental health conditions. It is estimated that about 10–20% of children and young people are diagnosed with a chronic illness [3,4]. The increase in chronic illness diagnoses is expected to rise further over the next few decades [4]. The rise could be caused by improved treatment, screening, and rehabilitation, which allow students to survive illnesses and engage with society [3]. Students with chronic illnesses, such as neuromuscular diseases, are at higher risk of experiencing poor educational outcomes due to hospitalizations, treatment, and symptoms such as fatigue and muscle weakness, which lead to increased school absences [5,6]. Consequently, targeted educational support is essential to enable these students to participate in school and complete their education [7,8,9]. School absence can be categorized as excusable or inexcusable [10,11,12]. Excusable school absence can be related to medical or illness reasons, while inexcusable school absence is related to social or environmental reasons [11,12]. In the Scandinavian countries, schools are required to provide support for students with chronic illnesses [13,14,15]. A traditional approach to securing education for students with chronic illnesses and school absences is through hospital schools and home instruction [8,13]. Another newer approach involves technological solutions, such as telepresence robots, that enable students to participate in education without being physically present at the school [16,17,18]. An example of a telepresence robot is AV1, which can be placed in the classroom, allowing students to connect to it through a tablet or smartphone (Figure 1) [19].

In the education environment, the student can see, speak, and hear through the telepresence robot AV1. The telepresence robot also has functions such as rotation and raise-a-hand, and it conveys emotions through its eyes [19]. The aim of the telepresence robot is to connect students with chronic illnesses to the education system, fostering a stronger sense of belonging and engagement [20,21]. Recent research on the use of the telepresence robot AV1 has investigated experiences in schools among teachers and students with different chronic illnesses [17,21,22,23]. Studies have investigated how telepresence robots can be used for students with different types of school absence, such as problematic absenteeism, hospitalization, treatment, side effects of chronic illness, or isolation due to the COVID-19 pandemic [17,23]. Examples of students who have used the telepresence robots in the education system include students with cancer, chronic fatigue syndrome, and school avoidance [22,24,25]. The telepresence robot has the potential to include students in social and learning activities [16,20,23,26]. Nevertheless, there are challenges and barriers to introducing telepresence robots in educational settings such as skepticism and a lack of guidelines to facilitate their use among teachers, students using the robots, and their classmates [17,20,25]. The integration of telepresence robots involves collaboration between several stakeholders such as teachers, parents, school principals, and students [27,28]. Therefore, to ensure better facilitation and implementation in the future, it is crucial to investigate how community care stakeholders approach the implementation and integration of telepresence robots for students with chronic illness and school absence within the Scandinavian education system.

## 2. Methods

### 2.1. Aim of the Study

The aim of this study was to investigate the reflections of Scandinavian community care stakeholders on telepresence robots as a tool to reduce school absence among students with chronic illnesses in the education system.

### 2.2. Methodological and Theoretical Framework

The design was a qualitative interview study using the interpretive description (ID) methodology, with Edgar Schein’s theory of organizational culture as the theoretical lens [29,30,31]. ID is an inductive qualitative methodology that can strengthen the description and interpretation of the research aim examined and thereby improve practice [29,30]. The ID methodology was used in this study to develop practice knowledge and identify experiences and perspectives on the use and implementation of telepresence robots to reduce school absences. To understand and analyze the complexity of the change process and relationships involved in using telepresence robots to reduce school absence in the Scandinavian education system, Edgar Schein’s three levels of organizational culture and leadership were applied [31]. The theory provides a structural and practical description of the relationships within an organization during periods of change. Schein’s culture framework is divided into three levels: 1. Artefacts and behaviors are elements that are visible and can be perceived by sight and touch. 2. Espoused values refer to the declared values, norms, and behaviors; they represent the less visible aspects of culture.; and 3. Basic assumptions are the unspoken rules we accept as normal ways of understanding the world and signify an unconscious aspect of culture [31]. The three levels of culture (Artefacts, Espoused values, and Basic assumptions) assisted the understanding and relationships of the behavior and challenges from the community care stakeholders’ experiences and perspectives in integrating telepresence robots in the Scandinavian education system [31].

### 2.3. Sampling

The participants were recruited and interviewed between 1 April 2025 and 30 September 2025. The sampling method was purposive and included community care stakeholders in municipalities who organized telepresence robots to reduce school absences in compulsory education. The inclusion criteria for the community care stakeholders were: 1. Working with more than two telepresence robots; 2. Organizing telepresence robots in compulsory education; and 3. Having experience in organizing telepresence robots to reduce school absence for students with chronic illnesses. We aimed to achieve maximum representation across the three countries (Norway, Sweden, and Denmark) in terms of municipality size, years of experience, location, and number of telepresence robots owned. The first author contacted municipalities in Denmark (n = 12), Norway (n = 11), and Sweden (n = 25) that were using telepresence robots to reduce school absence via email, providing information about the research project and inviting them to participate in individual or focus group online interviews. After three weeks, we contacted the municipalities that had not replied to the first email. We broadly identified community care stakeholders as individuals who worked across different municipal settings to organize the use of telepresence robots in the education system. Some stakeholders worked full-time on organizing the telepresence robots in the education system, while others also had other functions. Most of the stakeholders had backgrounds as teachers, special educators, or special needs teachers. Others had backgrounds as administrators or technical support staff in the municipalities’ educational departments. They all worked with inclusive education or special educational needs. In total, 25 community care stakeholders agreed to participate in an interview and were included in the study (see Table 1). The sampling included an overrepresentation of community care stakeholders from Sweden, as more Swedish municipalities use telepresence robots in education. The characteristics of the participants are described in Table 1.

### 2.4. Data Generation

The study was conducted within the context of the Danish National Rehabilitation Center for Neuromuscular Disease [32]. Data were generated through a combination of semi-structured interviews and semi-structured focus group interviews. Focus group interviews were applied when there was more than one community care stakeholder in a municipality. The focus group interviews enabled community care stakeholders to interact and discuss their perspectives in response to the interview’s questions. The same interview guide was used for both types of interviews. All interviews were conducted by the first author, who was considered an insider with expert practical and research knowledge of telepresence robots in the Danish education system [29,30]. Due to the distance between the three countries and the participants’ time constraints, the interviews were conducted online by the first author via Microsoft Teams. Participants joined the interviews from their municipal offices in Sweden, Norway, or Denmark. In total, fifteen semi-structured interviews and four focus group interviews with community care stakeholders were conducted (see Table 1).

The interview guide was translated into the three Scandinavian languages (Swedish, Norwegian, and Danish). The author interviewed the participants in their native language, and if any language barriers arose, the questions were asked in English. Translating the questions into the three languages strengthened the understanding. Schein’s culture framework guided the interview guide, aiming to understand community care stakeholders’ perceptions and the cultural and organizational changes related to telepresence robots and to reducing school absence in the education system [31]. Open-ended questions regarding the research aim were asked during the interview [32]. Examples of questions in the interview guide are listed in Table 2. In the focus group interviews, it was important to ensure that all the participants discussed and answered the questions in the interview guide. Based on Schein’s cultural framework, the follow-up questions addressed skepticism, communication, roles, and collaboration. The mean length of the interviews was 53 min [35 min to 75 min]. All interviews were audio-recorded and subsequently transcribed verbatim with the assistance of the app tool “Transcriber”(version 1.10) [33] on the UCloud platform accessible to Aarhus University researchers. The first author then checked the transcripts for errors.

### 2.5. Data Analysis

**Figure 2:**
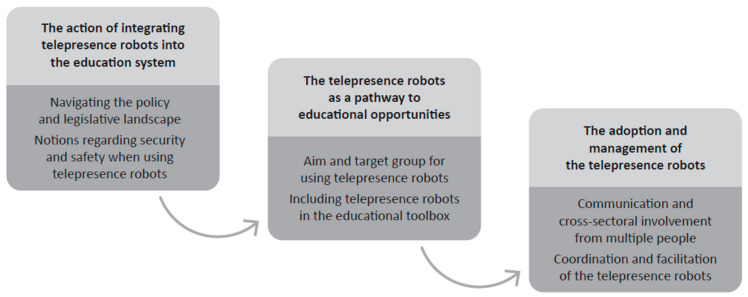
Reflections of the community care stakeholders’ experience with telepresence robots in the Scandinavian municipalities’ education system.

Data analysis was conducted by the first and last author in an iterative inductive four-step process following the ID methodology [29,30] (Table 3). The four steps were revised multiple times. In step 1, all data were transcribed and uploaded to the software program NVivo 14^TM^ (version 14.24.3) to organize and manage the data material. The author group individually reviewed key interviews, discussed their findings, and established initial codes aligned with the study aim around community care stakeholders’ perspectives on using telepresence robots. Then the first author did the initial coding of all the data. In the second step, the first author reread all the data and coded general patterns and relationships to create insights into the community care stakeholders’ perspective on using telepresence robots. The first and last authors discussed the coding to identify subthemes related to the study aim [29,30]. In step three, patterns and relationships were identified and discussed to determine themes and subthemes. The first author identified categorical themes based on subthemes and discussed the final themes to be determined. Afterward, all authors were presented with the subthemes and categories to discuss and finalize the categories [29,30]. In step four, the main messages were identified, leading to an interpretive thematic and conceptual overview of relationships and patterns among the themes, as well as an illustrative summary of the findings (Figure 2). The first author drafted the findings and the illustrative figure. The author group discussed the relationships and interpretations of the findings to qualify the analysis [29,30]. Disagreements regarding coding or interpretation of the findings were addressed through iterative discussion until consensus was reached. During the four steps of the ID analysis, the data were analyzed inspired by the theoretical lens of Organizational culture by Edward Schein [31]. Organizational Culture served as an inspiration, helping us to understand the implementation and change processes involved in integrating the telepresence robot into the education system by multiple people. Organizational culture influenced the creation of subthemes and categorical themes as well as the interpretation of relationships among these themes [31].

## 3. Results

The findings provided insights into the work and reflections of community care stakeholders regarding the inclusion of the telepresence robot as a tool to reduce school absences among students with chronic illnesses (Figure 2). The first theme, “The action of integrating telepresence robots into the education system,” entailed “navigating the policy and legislative landscape” and “Notions regarding security and safety when using telepresence robots.” The second theme “The telepresence robot as a pathway to educational opportunities” highlights “Aim and target group for using the telepresence robots”, and “Including the telepresence robots in the educational toolbox”. The third theme “The adoption and management of the telepresence robots” was influenced by “Communication and cross-sectoral involvement from multiple people” and “Coordination and facilitation of the telepresence robots”.

### 3.1. The Action of Integrating Telepresence Robots into the Education System

#### 3.1.1. Navigating the Policy and Legislative Landscape

The implementation process of telepresence robots was long and challenging, as ethical and data protection permissions needed to be in place before the implementation could begin. The community care stakeholders reflected on how they had to navigate various permissions to use the telepresence robots in the schools.

“I had to make a 20-page digital platform information report and risk analysis… So, it took a very long time. It was a bit frustrating because the school principal waiting for this robot. We had children who were registered as potential candidates. So, we had a huge delay there… Then we also had a discussion with the educational-psychological service because there are different opinions on school absence. What is working and what to do about it”

(Female, two years of experience with telepresence robots, Norway)

The quotation exemplifies how multiple stakeholders are involved in integrating telepresence robots into the education system, and critical perceptions among them delay the adoption and acceptance. Community care stakeholders reported that the education framework and compulsory education legislation (The Education Act) were barriers to the implementation of telepresence robots in schools. The legislation around compulsory education was not explicit on the use of telepresence robots in schools. The community care stakeholders explained how guidelines on ministerial education department websites in the three countries had been developed around the use of telepresence robots. The community care stakeholders described how they had participated in networks with other community care stakeholders in their countries and shared knowledge to navigate the legislative and political landscape.

“The legislation was written before this technology existed, and here the municipalities do things very differently. Should it be considered distance learning? No, then we don’t have permission for it. But we argue that, and so does our legal adviser, that the important thing is getting the student to school. It’s about working with attendance, not the teaching itself… So, the law is unclear, or it is a grey area. It depends on how you interpret the law”

(Male, five years of experience with telepresence robots, Sweden)

This quotation illustrates how the community care stakeholders were working on changing the foundation for using telepresence robots. Stakeholders expressed hope that the education legislation would evolve to benefit telepresence robots and adopt it as an accepted educational tool. The education departments had the autonomy to initiate new projects. Community care stakeholders informed education departments and decision-makers about telepresence robots, who were generally supportive of their use in schools to reduce school absence. Community care stakeholders played a crucial role in communicating and advocating for the adoption and acceptance of telepresence robots in the municipalities. Furthermore, they possessed a comprehensive understanding of the legislation, data protection, and ethical considerations, enabling them to effectively navigate and guide their education department toward the appropriate use of telepresence robots in schools. Introducing new artefacts to reduce school absence for students with chronic illness represented new opportunities to attend education. Moving beyond contrasting perceptions, negotiating assumptions about educational support appeared to be a crucial step which brought all three levels of culture into play.

#### 3.1.2. Notions Regarding Security and Safety When Using Telepresence Robots

Communication about security and integrity was described as a core element in securing the acceptance of telepresence robots in schools. The discussion about security exposed an underlying assumption that technology is dangerous in the education system. The community care stakeholders noted that the schools were initially skeptical about installing a camera in the classroom and livestreaming to students at home or to another classroom at the school. Some community care stakeholders described how they communicated with the school’s principal, teachers, parents, and students about the safety of telepresence robots. The approval procedure for using the telepresence robot in the classroom varied across schools. The community care stakeholder noted that some schools required parents to provide written consent confirming that only the student would use the telepresence robot. Some schools also required parents of other students to consent to the use of the telepresence robot in the classroom. Most community care stakeholders explained that all teachers had to sign a declaration. At other schools, informing about the telepresence robots was enough. Most community care stakeholders described how specialized guidelines had been developed for students; for example, students were required to use headphones and remain in their rooms when using telepresence robots. This also included guidelines prohibiting students from recording lessons and prohibiting parents from being in the same room when students used telepresence robots during the school day.

“So, there is nothing digital in the system that says that this student has this robot. And it’s also not possible to record anything. There is nothing that remains for the next step. So, it seems as if the company has thought about it from a GDPR point of view, how to use it. It locks up immediately if you’re trying to record, for example.”

(Male, five 5 years of experience with telepresence robots, Sweden)

This quotation illustrates how community care stakeholders continued to argue for and explain the functions of the telepresence robot to reduce negative assumptions and critical reflection in the education system. The community care stakeholders took time to explain about the security and use of telepresence robots at introductory meetings with teachers. The teachers had many questions about livestreaming in the classroom, the non-facial telepresence robot, and how to respond in different teaching situations when a student was following the teaching through the telepresence robots. The community care stakeholders reflected on how unfamiliar the technology was to teachers and on their fear of what the telepresence robots would do in the classroom. This room for sharing reflections and knowledge appeared relevant to establish common ground for using telepresence robots in the education system. Skepticism often arose at the beginning of the implementation of the telepresence robot, before the introductory meeting, as teachers were still inexperienced with using them in the classroom. There was an ambivalence in using telepresence robots in the education system, manifesting as a resistance to change, on one hand, and recognizing potential benefits on the other hand

“This alien-like thing, you know, when you see it there, lighting up and all, I’m thinking it’s going to pollute the classroom. So, I think it is kind of… And maybe there’s also still this narrative that the robots are coming to take over our teaching and stuff like that.”

(Male, 2 years of experience with telepresence robots, Denmark)

The quotation exemplifies how underlying assumptions around telepresence robots were negotiated through critical reflection and perceptions. The stakeholders acknowledged the teachers’ concerns and collaborated with them to explore the potential of telepresence robots to support a student’s return to education or re-entry into school after a period of absence. By communicating about underlying assumptions, the understanding of telepresence robots among multiple people attendees was more positive, and the benefits were discussed instead.

### 3.2. Telepresence Robots as a Pathway to Educational Opportunities

#### 3.2.1. Aim and Target Group for Using Telepresence Robots

Expanding educational support practices to include telepresence robots may enhance participation for students with chronic illnesses in the education system. The community care stakeholders described how school absence had been a concern in their municipalities over the past couple of years. Most municipalities invested in telepresence robots following the COVID-19 pandemic, driven by additional project funding and increased student absence rates in compulsory education and upper secondary education. The participants explained the need to provide education to students with school absence and emphasized that the aim of telepresence robots was to establish social and academic connections with the school. At the level of espoused values, the use of telepresence robots reflects organizational intentions related to inclusion, equal educational opportunities, and social belonging. The telepresence robots serve as a visible manifestation of the education system’s attempts to address school absence. The community care stakeholders identified two groups that could benefit from telepresence robots to reduce school absenteeism in their municipalities. The first group consisted of students with chronic illnesses who could not attend school due to physical circumstances such as hospitalization or treatment in their homes. The participants had experienced good results with this group. Most community care stakeholders described students with chronic illnesses as well integrated into the classroom and maintaining good relationships with their teachers. The telepresence robots provided students with chronic illnesses access to the school, allowing them to follow classes and connect socially with their classmates. Here, a community care stakeholder outlined how telepresence robots could be used as a strategy to support students with chronic illness in taking on a more active role in their education

“When it comes to illness, the telepresence robot is introduced a little sooner… And the student may be more motivated too. I don’t want to get behind in school. I want to see my classmates because I feel very isolated when I can’t be in the classroom. In those cases, it feels a bit more student motivated. The desire to stay connected with the school”

(female, one year of experience with telepresence robots, Sweden)

This quotation illustrates how the purpose of the telepresence robots is connected to the individual needs of students with chronic illnesses. Most of the community care stakeholders described a second group identified as students with problematic school absence and for example, diagnoses like ADHD and autism. Some participants described this group as not particularly motivated for returning to school and as having been away from school for an extended period. Students with problematic school absence used telepresence robots to observe the classroom and reconnect with the school after an absence period. A community care stakeholder explained how the telepresence robot could also provide opportunities to include students with problematic school absences.

“For the students with problematic school absence, our goal is that they remain in school; they have so much absence from school. Here, the telepresence robot can give them a sense of belonging to the class again and make sure they are taught, because they haven’t been taught for a long time”

(Female, 2 years of experience with telepresence robots, Denmark).

This quotation exemplifies how the telepresence robot can have various purposes depending on the target group. The community care stakeholders considered how the telepresence robot could benefit different groups of students and how its use could expand in the future. In summary, the two identified groups used the telepresence robot for different purposes yet shared the same overall aim: reconnecting with the school.

#### 3.2.2. Including Telepresence Robots in the Educational Toolbox

The possibilities of adopting telepresence robots into the educational toolbox as part of a combined approach with other solutions were highlighted. Telepresence robots were described as a new tool in the toolbox for students with chronic illnesses with school absence. The community care stakeholders explained how they adapted and integrated telepresence robots to meet the specific needs of each student struggling with school absence. The participants reflected on how an early intervention to prevent school absences could be facilitated using telepresence robots. A participant highlighted how more solutions for reducing school absences were needed in the future in the education system.

“However, when working with school absence and students who are unable to attend school, it is essential to have as many tools as possible in your bag. And a telepresence robot, could be a really good tool”

(Male, 2 years of experience with telepresence robots, Denmark)

The telepresence robots were still a relatively new educational tool and adapting them into the toolbox was a time-consuming process. At the level of underlying assumptions, the education system was still influenced by traditional views of participation, and learning took place in the physical classroom. Most community care stakeholders noted that the first step in implementing telepresence robots in schools was to secure their adoption as a tool to reduce school absences. From a future perspective, some participants expected telepresence robots to be available in schools for early intervention and day-to-day use. The stakeholders described situations in which telepresence robots could be valuable, such as placing students who need a break in another room or reducing school absences for students who cannot be present for a few days. An experienced community care stakeholder explained that telepresence robots are versatile tools and adaptable to the various needs of students:

“We’ve had students who can manage to spend some part of the day in the class but can’t manage to stay there because they become overstimulated and their energy runs out. And then it’s been good to have a robot for the rest of the day. Yes. So, we’ve used them for different purposes”

(Female, five years of experience with telepresence robots, Sweden)

The quotation illustrates how community care stakeholders see opportunities for telepresence robots in the education system, their flexibility and a new approach to educational participation for students being absent from school. A broader acceptance of the telepresence robot may depend on changing underlying assumptions about educational support and participation in the education system. In summary, people in the education system found adapting the telepresence robot and its many opportunities time-consuming and difficult to accept. More knowledge sharing about the use of a telepresence robot may reduce uncertainty and highlight the benefits among multiple people.

### 3.3. The Adoption and Management of the Telepresence Robots Communication and Cross-Sectoral Involvement from Multiple People

Expanding the use of telepresence robots in the education system requires an understanding of their potential as a tool for education. Community care stakeholders reflected on communication strategies regarding telepresence robots across organizational levels within municipalities. They explored ways to change perceptions and perceived values of telepresence robots as a tool to compensate for school absence. The participants communicated on various platforms about the telepresence robots; some shared information on the local intranet and others participated in local networks to inform teachers. The community care stakeholders noted that they needed to collaborate with multiple people to adapt telepresence robots to the school context.

“Everybody who works with children and young people know that this is a tool we have. So, we are trying to communicate to them that here is a relevant tool that’s useful for them, when they are involved in such cases, and that they should know about it.”

(Male, five years of experience with telepresence robots, Norway)

This quotation illustrates the difficulty of reaching all the people who need to know about telepresence robots, which may be a barrier to adoption in the education system. Most community care stakeholders reported that school principals played a significant role in the adoption of telepresence robots. They pointed out that a positive attitude toward telepresence robots among school principals influenced teachers’ desire to incorporate them into the schools. Furthermore, the school principals were often the primary point of contact between community care stakeholders and teachers.

“It is also the school principals who are responsible for the students, and it is the principals who ultimately communicate with you, the community care stakeholder, maybe. Or with a hospital teacher. Or with guardians, students, and so on. Because the school principals are primarily responsible for the students.”

(Male, three years of experience with telepresence robots, Sweden)

The quotation highlights how communication with school principals and changes in behavior or values in cases of school absence were essential. The participants explained that the work was cross-sectoral and involved communication with teachers, school principals, and, in some cases, special-needs teachers and psychologists. Parents and students also needed information and support to foster willingness and motivation to use telepresence robots. Community care stakeholders often felt misunderstood by school principals and teachers during the introduction of telepresence robots. There were numerous interpretations surrounding the telepresence robots, and stakeholders were compelled to clarify their communication. Cross-sectional collaboration can be challenging, as the people involved have different ways of perceiving, thinking, and feeling regarding the school absence problem. The community care stakeholders play a vital role as leaders in the change process of integrating telepresence robots into the education system.

#### Coordination and Facilitation of the Telepresence Robots

Community care stakeholders had used telepresence robots for varying lengths of time. Some stakeholders had many years of experience with implementing telepresence robots in schools. In contrast, others had recently begun working with telepresence robots and were in the early stages of introducing them to schools. Most participants described the implementation of telepresence robots in the school as a ‘learning by doing’ process. Most community care stakeholders reported that the implementation process began when a school principal applied to the central municipal education department for a telepresence robot for a student with frequent school absences. An experienced community care stakeholder explained how the implementation process of a telepresence robot works.

“You apply to us if you have a student with different types of absence, then you send an application to the central student health department, which is us. Then we examine whether everything has been done, what needs to be done, that the student has, like… that you’ve tried different measures before you go as far as a telepresence robot”

(Female, seven years of experience with telepresence robots, Sweden)

The quotation outlines how schools are responsible for recognizing and appreciating the benefits of the telepresence robot in cases of school absence. When a school applied for a telepresence robot, the participants organized a meeting where they set up and connected the telepresence robot to the tablet and introduced its functions. The introductory meetings also addressed safety concerns, usage times, and how to structure the school day with a telepresence robot.

“First, I want to meet with the staff at the school, and then I usually meet with the teachers who have the class where the robot will be used…. So that you control things and help them discuss what is best for this school—and then inform them about how to document it in the student’s action plan”

(Male, six years of experience with telepresence robots, Sweden)

This quotation represents the practical approach to integrating the telepresence robot into the education system. Here, the implementation process is organized and structured around a meeting in which assumptions and behaviors regarding the telepresence robots are discussed. Community care stakeholders received positive feedback during and after the introduction meeting. They described the importance of being available to and supportive of schools and teachers during the implementation process. The participants noted that a structured approach to using a telepresence robot was appreciated by the teachers. Moreover, the use of telepresence robots depended on the students’ motivation, which appeared stronger when schools developed their own solutions and organizational approaches for deploying telepresence robots among students.

In summary, skepticism and ambivalence about adopting telepresence robots appeared to stem from the artefact level of reasoning as defined by Schein. By communicating and negotiating assumptions about telepresence robots within the education system, all three levels of culture (artefacts, espoused values, and basic assumptions) were engaged, enabling shared perceptions to form. The community care stakeholders played a leading role in the change process of adopting and accepting the telepresence robot in the education system.

## 4. Discussion

There is limited knowledge of community care stakeholders’ reflections on telepresence robots in municipalities in a Scandinavian context. In the present study, we found that community care stakeholders in municipalities are recognizing technological solutions as supplements to traditional educational support to reduce school absence for students with chronic illnesses. Previous research has primarily focused on how parents, teachers, and students with chronic illnesses experience challenges and barriers in navigating the education system [2,7,34,35]. Students with chronic illnesses often experience a negative impact on social interaction and academic performance within the education system [2,8,36]. According to a Danish report, schools often fail to provide the necessary educational support for students with chronic illnesses, preventing them from participating in their education [37]. Without the necessary educational support, this has negative consequences for the social, academic, and emotional needs of students with chronic illnesses [2,8]. This present study showed that telepresence robots were an additional tool for managing school absences, primarily for students with chronic illnesses. Most studies in the field of telepresence robot research have focused primarily on students with chronic illnesses such as cancer, neuromuscular diseases, and chronic fatigue syndrome [17,25]. In our study, telepresence robots were included in the educational toolbox for students with school absences due to chronic illness to ensure continued participation in education. Access to education can be particularly challenging for students with chronic illness, as frequent absence from school due to treatment, illness, or other health-related consequences often disrupts their educational participation [8]. According to community care stakeholders, telepresence robots can facilitate reconnection to and participation in the school environment for students with school absences. Increasing awareness of telepresence robots as an educational tool could be crucial to moving the underlying assumptions about participation in education. Telepresence robots are artefacts in the education system, serving as visible tools for engaging with technology and supporting inclusive participation and learning in the municipality.

The community care stakeholders in the present study were pioneers in fostering the adoption and implementation of telepresence robots as an educational tool to reduce school absences and reconnect students with the education system. They reflected on the long and challenging process of integrating telepresence robots into the education system and felt they needed to navigate legislation, political decisions, and various permissions before telepresence robots could be introduced. A previous study found that Scandinavian education legislation provided only sparse information and guidelines on the use of telepresence robots for children with chronic illness and school absence [13]. The sparse legal information might explain why the participants in the present study were navigating a grey area when integrating telepresence robots into the education system in the Scandinavian setting. To improve the integration of telepresence robots in the education system, it is essential to develop general guidelines and information for teachers, school principals, and other stakeholders. Our findings showed that community care stakeholders often experienced skepticism from teachers and school principals when trying to integrate telepresence robots into the education system. Previous studies have shown that implementation can be challenging, as telepresence robots remain relatively new and unfamiliar in the education system [17,21,38]. Especially, teachers express skepticism and concerns about using telepresence robots in their classrooms, particularly regarding security and privacy [17,38]. At the level of artefacts, current school practice often positions physical presence as the norm for participation, and organizational structures are not designed to accommodate students who attend virtually [2]. Therefore, the use of telepresence robots’ challenges established practice and requires visible changes in how participation and operations are conducted in the education system. The participants in our study were aware of the skepticism and concerns and addressed them by providing information about telepresence robots to multiple people, including teachers, social workers, and school principals. Other studies highlight that parents were involved in convincing schools and teachers to give telepresence robots a chance in the classroom [39]. This highlights the importance of establishing clear guidelines for the use of telepresence robots in the Scandinavian education system to ensure that schools and teachers share similar underlying assumptions and values around the use. Further, it is essential that teachers and schools feel safe and confident using telepresence robots.

Our findings showed that introducing telepresence robots into the education system requires not only technical integration but also management and communication with multiple people to ensure alignment of expectations and responsibilities. Community care stakeholders played an essential role in integrating and implementing telepresence robots in the education system. Previous studies underscore the need for clear expectations and cooperation between parents and the school, for instance, regarding when and how long they participate, and how schools adjust instruction for the use of telepresence robots in classroom settings in the education system [21,22]. In this study, telepresence robots are centrally located within the municipalities’ education departments and deployed in schools by community care stakeholders. Other studies report a bottom-up approach in which parents or other individuals outside the education system provide information and engage with schools about the use of telepresence robots [23,39]. From Schein’s perspective, organizational change challenges the underlying assumptions of those affected. By integrating the findings with Schein’s framework, the study demonstrates that implementation of telepresence robots depends not only on access to technology but also on organizational willingness to redefine norms and assumptions around participation in educational practice [31]. Furthermore, the community care stakeholders are leaders and vital in ensuring the cultural change process and a safe transition to new behaviors and artefacts within the organization [31]. Accordingly, change processes are time-consuming and require an adoption period during which the people involved in the transition accept the new technology [31]. The study underscores the value of community care stakeholders’ working with the implementation and organization of telepresence robots to support students with chronic illnesses and reduce school absence in the education system.

### Methodological Considerations

Our study sample comprised community care stakeholders from various countries, positions, and backgrounds. The participants had varying levels of experience and different amounts of telepresence robots available to them. We consider the diversity of representation a strength, as we sought to identify patterns and relationships regarding the use of telepresence robots to reduce school absence in municipalities. We were interested in the organization and constellation of factors underlying the municipalities’ use of telepresence robots in Scandinavia.

The sample comprises more community care stakeholders from Sweden than from Norway or Denmark. It is our impression that investment in telepresence robots in Sweden has increased over the past couple of years [19]. Only a few municipalities in Norway and Denmark used telepresence robots in their schools, which hindered recruitment of additional participants in these two countries. Even though it could be argued that the overrepresentation of Swedish community care stakeholders has influenced the findings, our intention was not to compare the countries but to identify patterns and relationships in the integration of telepresence robots in the education systems across Scandinavia. The Scandinavian countries share many structural and cultural characteristics in their education systems. The community care stakeholders provided relevant and rich information about their experiences, which allowed us to uncover new knowledge about the work of community care stakeholders on implementing telepresence robots in the three countries’ education systems [29,30].

The first author had extensive experience in the field of telepresence robots, which strengthened the interview questions and enhanced the understanding of the reflections from the community care stakeholders’ perspective. Conversely, this preunderstanding might have restricted the questions asked during the interviews. The first and last author read the transcripts separately and later discussed the findings and interpretations in accordance with the ID methodology’s analysis process (See Table 3) [29,30]. The involvement of multiple researchers ensured research triangulation through diverse interpretations and perspectives in the analysis process [29,30]. In our study, the use of the organizational culture levels methodology provided insights into the underlying assumptions and values when integrating a telepresence robot into the education system [31]. The ID methodology is inductive, and Schein’s organizational theory informs the analysis, results, and discussion of the findings [29,30,31]. Our findings should be transferable to similar settings and contexts involving telepresence robots in other countries’ education systems. Future studies should explore the political implications of using a telepresence robot as an educational tool to reduce school absence.

## 5. Conclusions

The study provided new insights into the challenges faced by community care stakeholders when integrating telepresence robots into the Scandinavian education system for students with chronic illness and school absence. The findings illustrated the complexity of adopting telepresence robots into the education system in Scandinavia. Awareness of telepresence robots as educational support tools in Scandinavian education systems is increasing, and recognition among community care stakeholders is reshaping assumptions about the use of technology for education. Community care stakeholders were leaders in driving organizational change to adopt and accept the use of telepresence robots in the education system. They had to navigate multiple people in the education system and possess knowledge of the technology, implementation, legislation, and permissions. Furthermore, community care stakeholders had to communicate with and support the schools in using telepresence robots to ensure smooth implementation. This study provides important insights into the implementation of telepresence robots in the Scandinavian education system. The study offers contextual knowledge that may be transferable to similar educational systems and contexts with comparable organizational, cultural, and policy conditions. Further research across diverse national and institutional contexts is needed to examine the broader transparency of telepresence robot implementation in education systems beyond Scandinavia.

## Figures and Tables

**Table 1:** Participant description.

Gender of the Participants n (%)	Community Care Stakeholders (n = 25)
Female	15 (60%)
Male	10 (40%)
**Years of experience with telepresence robots, n (%)**	
1–3	12 (48%)
4–6	10 (40%)
6–8	3 (12%)
**Age at the interview (median, range)**	48.5 (38–57 years)
**Number of participants per country, n (%)**	
Sweden	15 (60%)
Denmark	5 (20%)
Norway	5 (20%)
**Number of telepresence robots per municipality (median)**	14.55 (2–85)

**Table 2:** Examples of questions in the semi-structured and focus group interview guide.

What is the aim of using the telepresence robots in education? Why did you make the decision to invest in telepresence robots? How does the legislation describe the use of telepresence robots in a school context? How was the process of getting permission to use telepresence robots in the municipalities? Who is in the target group for telepresence robots? How do you organize the telepresence robots in your municipalities? How did the school react to the introduction of the telepresence robot? Which political/strategic goals have you set for the telepresence robot? Which guidelines for the use of telepresence robots have been prepared for the schools in your municipalities? How are the telepresence robots integrated into the schools’ work with school absence? What are the future aspects of the use of telepresence robots in your municipality?

**Table 3:** Illustration of the analysis and coding process guided by the Interpretive description methodology.

	First Step	Second Step	Third Step	Fourth Step
Description of the Analytical Step of Interpretive Description	Initial Coding	Generalized Patterns	Final Categories	Illustration Visualizing the Final Findings
Codes and subthemes leading up to the final categorical themes	The aim of the telepresence robots Working with the telepresence robots in the organization User groups for the telepresence robots	Navigating the policy and legislative landscape Notions regarding security and safety when using telepresence robots	The action of integrating telepresence robots into the education system	Figure 2
	Legislation and policy for using the telepresence robots Complexity when using telepresence robots Safety and permission using telepresence robots	Aims and target group for using telepresence robots Including telepresence robots into the educational toolbox	The telepresence robots as a pathway to educational opportunities	
	Organization of the telepresence robots Collaboration with school Different person involved in using the telepresence robots Coordination and facilitation	Communication and cross-sectorial involvement from multiple people Coordination and facilitation of the telepresence robots	The adoption and management of the telepresence robots	

## Data Availability

The datasets generated and/or analyzed during the current study are not publicly available due to privacy and ethical restrictions, but are available from the corresponding author on reasonable request.
